# Potential of Sodium MRI as a Biomarker for Neurodegeneration and Neuroinflammation in Multiple Sclerosis

**DOI:** 10.3389/fneur.2019.00084

**Published:** 2019-02-11

**Authors:** Konstantin Huhn, Tobias Engelhorn, Ralf A. Linker, Armin M. Nagel

**Affiliations:** ^1^Department of Neurology, Friedrich-Alexander-University of Erlangen-Nuremberg, Erlangen, Germany; ^2^Department of Neuroradiology, Friedrich-Alexander-University of Erlangen-Nuremberg, Erlangen, Germany; ^3^Department of Neurology, University of Regensburg, Regensburg, Germany; ^4^Department of Radiology, Friedrich-Alexander-University of Erlangen-Nuremberg, Erlangen, Germany; ^5^Division of Medical Physics in Radiology, German Cancer Research Center (DKFZ), Heidelberg, Germany

**Keywords:** multiple sclerosis, magnetic resonance imaging, sodium MRI, ^23^Na MRI, neurodegeneration, biomarker

## Abstract

In multiple sclerosis (MS), experimental and *ex vivo* studies indicate that pathologic intra- and extracellular sodium accumulation may play a pivotal role in inflammatory as well as neurodegenerative processes. Yet, *in vivo* assessment of sodium in the microenvironment is hard to achieve. Here, sodium magnetic resonance imaging (^23^NaMRI) with its non-invasive properties offers a unique opportunity to further elucidate the effects of sodium disequilibrium in MS pathology *in vivo* in addition to regular proton based MRI. However, unfavorable physical properties and low *in vivo* concentrations of sodium ions resulting in low signal-to-noise-ratio (SNR) as well as low spatial resolution resulting in partial volume effects limited the application of ^23^NaMRI. With the recent advent of high-field MRI scanners and more sophisticated sodium MRI acquisition techniques enabling better resolution and higher SNR, ^23^NaMRI revived. These studies revealed pathologic total sodium concentrations in MS brains now even allowing for the (partial) differentiation of intra- and extracellular sodium accumulation. Within this review we (1) demonstrate the physical basis and imaging techniques of ^23^NaMRI and (2) analyze the present and future clinical application of ^23^NaMRI focusing on the field of MS thus highlighting its potential as biomarker for neuroinflammation and -degeneration.

## Sodium and the Pathophysiology of Multiple Sclerosis (MS)

As a widely accepted paradigm, the pathology of MS is hallmarked by inflammatory demyelination but also neuro-axonal damage. In fact, neurodegeneration occurs already at the early stages of the disease constituting a primary contributor to sustained or progressive disability in the longer disease course (1, 2). On a cellular level, the maintenance of a transmembrane ion gradient resulting in a negatively charged intracellular and positively charged extracellular space is crucial to enable vital electrochemical signal transduction in humans. This process strongly depends on sodium: the ion gradient is largely created and maintained by the energy consuming Na^+^/K^+^-ATPase, leading to cellular efflux of 3 Na^+^ ions and influx of 2 K^+^ ions. Independent of its origin, loss of ATPase function leads to breakdown of the resting transmembrane potential difference, intracellular sodium accumulation, deficiency of the ATP generating mitochondrial respiratory chain and finally to expiring signal transduction and cell death as well as increase of the extracellular volume fraction (3, 4).

In MS, chronically demyelinated axons are prone to degeneration and trophic failure as consequence of an increased energy demand. Maladaptive repair and neuro-axonal rearrangements as well as a decreased ATP supply may further contribute to this process. Finally, lack of energy may lead to breakdown of the Na^+^/K^+^-ATPase as a major energy consumer in the CNS (5). In fact, chronically demyelinated MS lesions display a substantially reduced axonal Na^+^/K^+^-ATPase expression (6).

Additionally, the pivotal role of pathologic sodium accumulation in MS was previously demonstrated in several *ex vivo* and *in vivo* studies (7). These studies point to a compensatory redistribution or over-expression of distinct voltage-gated Na^+^ channels (e. g. Nav1.2, Nav1.6) on demyelinated axons in order to compensate for demyelination. This is an energy demanding process that is hardly sustained in already energy deprived axons. Thus, energy failure and toxic sodium accumulation may initiate a vicious cycle. Consecutively, increased intracellular sodium concentrations may provoke reverse action of the Na^+^/Ca^2+^ exchanger and thus calcium accumulation, which leads to activation of neurodegenerative signaling cascades (8–11). Hence, application of therapeutic Na^+^ channel blockers like amiloride, lamotrigine, phenytoin, or carbamazepine display some neuroprotective properties in experimental MS models (12–16). However, clinical trials with Na^+^ channel blockers in MS are few and report conflicting results on potential neuroprotective properties, yet (17–19).

In addition, the so-called fat- and salt- (NaCl) rich “western-diet” has recently been implicated in the etiology of MS (20, 21). In this context, sodium reappeared in the center of (experimental) MS studies as a mediator of pro-inflammatory effects (21, 22). In cell culture, an excess of NaCl up to 40 mM led to enhanced pro-inflammatory Th17 cell differentiation. In experimental autoimmune encephalomyelitis (EAE), an animal model for MS, a high salt diet was associated with increased disease severity mediated by enhanced levels of pro-inflammatory Th17 cells (23). However, transfer from experimental to clinical studies has been difficult and results of clinical studies on the influence of sodium to MS are conflicting: In a first study, high salt intake was partly associated with disease activity (24). Yet, a retrospective analysis of a large interferon-beta treated cohort with clinically isolated syndrome (BENEFIT study) showed no relevant association of further disease activity with blood or urine sodium levels (25). Similarly, a study investigating MS with early onset failed to demonstrate an association of relapse activity and the amount of dietary salt intake (26). However, the retrospective nature of sodium exposure analysis and the lack of standardized sodium load quantification limit the definite validity of these studies.

In consequence of these conflicting study results and with the advance of sodium magnetic resonance imaging (^23^NaMRI) techniques, sodium MRI drew growing interest for the analysis of pathologic sodium accumulation and its consequences in MS. However, the history of sodium MRI application in the field of MS is short, only comprising about a decade to date. In addition to regular proton based MRI, ^23^NaMRI with its ability to measure brain sodium *in vivo* along with additional advantages of modern imaging techniques may constitute a promising biomarker for the influence of sodium on neurodegeneration and -inflammation in MS and vice versa. In our review, we aim at (1) demonstrating the physical basis and imaging techniques of sodium MRI and at (2) analyzing previous and future clinical applications of sodium MRI in the field of MS.

## Physical Basics and Imaging Techniques of Sodium MRI

Conventional MRI is based on the signal of protons (hydrogen, H^+^). Protons exhibit the best properties for *in vivo* MRI due to their large gyromagnetic ratio and their high abundance in human tissues, predominantly contained in water or fat (27, 28). Besides protons, MR imaging of other so called “X-nuclei” is feasible in principle if they inhere a non-zero nuclear magnetic spin moment, which requires an odd number of protons or neutrons (29). Almost all elements of the periodic table have at least one isotope that fulfills this requirement (30). However, the most limiting issue for X-nuclei MRI is the signal-to-noise ratio (SNR), which is proportional to the *in vivo* concentration, the physical MR sensitivity of the nucleus and the voxel volume. For most isotopes either the physical MR sensitivity or the *in vivo* concentration is too low to achieve sufficient SNR and reasonable voxel volumes. Thus, only a few X-nuclei, such as oxygen (^17^O) (31), fluorine (^19^F) (32), phosphorous (^31^P) (33), chlorine (^35^Cl) (34), potassium (^39^K) (35, 36) and especially sodium (^23^Na) (37–39) have been used for MR imaging so far.

Among these, Na^+^ exhibits the best properties for *in vivo* MRI (40, 41).

Yet, ^23^NaMRI is challenged by low tissue sodium concentrations and an approximately 4-fold lower gyromagnetic ratio of sodium as compared to protons. For brain white matter, these shortcomings result in a roughly 5.500 times lower *in vivo* SNR of ^23^NaMRI vs. ^1^HMRI, if the similar acquisition times and voxel sizes would be used (41). Furthermore, Na^+^ highly interacts with surrounding macromolecules resulting in short biexponential T2 times (fast: 0.5–8 ms; slow: 15–40 ms; T1 time: 30–40 ms) (4, 42–45). To achieve sufficient SNR, only images with low spatial resolution can be acquired, which results in partial volume effects. In addition, longer acquisition times can be used to increase SNR. These effects limit the application of ^23^NaMRI. Additionally, distinction of different sodium compartments, i.e., intra-/extracellular, is difficult (29, 46–48).

Hence, dedicated acquisition techniques and elaborated post-processing may help to improve ^23^NaMRI imaging technologies. Above all, application of ultra-short echo-time (UTE) sequences is the common basis of quantitative sodium MRI (29, 49).

Additionally, ^23^NaMRI requires dedicated hardware, such as an appropriate radiofrequency (RF) amplifier and RF coils. Yet, both hardware components are not standard on routine clinical MRI scanners (50). Optimized scanner hardware may further improve the detection of the weak ^23^NaMRI signal. In detail, special dual-tuned ^23^Na/^1^H head array coils with up to 32 multi-channel receive arrays enable a synchronized registration of proton and sodium images with sufficient SNR (51–53).

As reviewed elsewhere, specialized ^23^NaMRI acquisition techniques, image reconstruction and post-processing techniques further improve SNR and spatial resolution. At the same time they reduce partial volume effects as well as acquisition time. Such techniques comprise compressed sensing with iterative reconstruction, sampling density weighted apodization (SDWA), twisted projection imaging (TPI), density-adapted 3D projection reconstruction (DA-3DPR), multi-echo radial sequences or different trajectories, i.e., 3D cones (29, 54–62).

**Figure 1:**
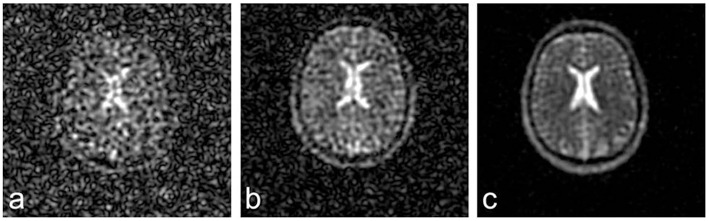
^23^NaMRI at 1.5 **(A)**, 3 **(B)**, and 7 T **(C)**. Similar acquisition parameters and a nominal spatial resolution of (4 mm)^3^ were applied. SNR increases approximately linearly with magnetic field strengths. Figure reproduced from (63) with permission of John Wiley and Sons, Journal of Magnetic Resonance Imaging.

In principle, sodium MR imaging is feasible at any magnetic field strength. However, due to the physical properties of Na^+^, application of at least 3 Tesla (T) field strength is warranted for a sufficient SNR and resolution. Modern 7 or 9.4 T ultra-high field MRI scanners further reduce the limitation of low signal strength, resulting in higher SNR (Figure 1) or improved spatial resolution (63). Nowadays, modern techniques enable sodium MRI of human brain at nominal spatial resolutions of 1 × 1 × 5 mm3 to 4 × 4 × 4 mm^3^ within 10–35 min acquisition time (48, 57, 64–67). Along with the development and advantages of high-field MRI scanners, research in the field of ^23^NaMRI has been prospering within the last decade without signs of any harm to study participants (51, 66, 68, 69). Comparing ^23^NaMRI signal intensities of brain tissue regions of interest (ROI) to control tubes containing predefined liquid saline solutions (i.e., 0–150 mM) placed beside the patient's head enables quantification of sodium concentrations (66).

MRI total tissue sodium concentrations (TSC) are the volume-weighted average of respective intracellular (normal: 10–15 mmol/l) and extracellular (normal: 140–150 mmol/l) sodium compartments. Typical intracellular volume fractions are on the order of 80% and extracellular volume fractions are around 20% (70). This leads to a TSC of ~40 mmol/l, which is close to results of studies directly analyzing the sodium concentration in brain white matter (3, 4, 47, 48, 71).

While classic ^23^NaMRI sequences only allow for quantification of TSC, differentiation between sodium accumulation in extracellular and intracellular compartments is even more interesting. Yet, the single resonance spectrum of sodium ions limits such discrimination. Use of paramagnetic shift reagents, which cannot pass cell membranes like anionic complexes of dysprosium or thulium, principally enables discrimination of intra- and extracellular sodium. Thus, MRI may detect shift of resonance lines exclusively in the extracellular space. However, clinical application of these compounds is not readily feasible for CNS studies due to their inability to cross the blood brain barrier and possible toxic effects (29, 72–74).

Instead, the application of relaxation-weighted imaging may be better suited for human studies. Preclinical studies showed that intracellular sodium exhibits shorter relaxation times (75). Thus, inversion recovery imaging (IR) can be utilized to suppress signals originating from sodium with a distinct T1 relaxation time. This may enable a weighting of the signal toward the intracellular compartment (29). At the same time, suppression of the CSF sodium signal also reduces disturbing partial volume effects when analyzing brain regions close to the CSF (71, 76–78). Sodium MRI IR sequences with a specific suppression of Na^+^ signals are comparable to the established fluid-attenuated-inversion-recovery (FLAIR) sequence of proton based MRI.

Sodium MRI IR sequences may be also the basis for the calculation of (pseudo) intracellular sodium concentrations and extracellular volume fractions (79). If additional anatomical masks from proton MRI are used, these techniques may even discriminate between intracellular sodium of white and gray matter (4). However, IR techniques only enable indirect calculation of compartmental sodium concentrations and are susceptible for confounders. In detail, the required intra- and extracellular relaxation times need to be estimated from preclinical studies and cannot be measured directly in humans, which might introduce a potential bias. In addition, relaxation times in pathologic structures are unknown and altered relaxation times may affect quantification. In consequence, some authors prefer the terms “pseudo-”intracellular sodium and “pseudo-”extracellular sodium for such analyses (79).

Alternatively, the use of two or more excitation pulses along with multiple quantum filtering (MQF; usually triple quantum filters = TQF) may also facilitate sodium compartment differentiation (80–82). In principle, the T2 relaxation based MQF allows for separation of different signals from sodium ions due to their variably restricted mobility within different compartments (81–86). However, MQF are prone to artifacts caused by field-inhomogeneity, low SNR or long acquisition times and its indirect calculation of sodium concentrations, similar to IR techniques (87). Recent quantitative multicompartment-multipulse techniques aim at exploiting differences in T1 and T2 relaxation times of different sodium compartments. This approach may enable separation of intracellular, extracellular and cerebrospinal fluid (CSF) signals, but is still hampered by low SNR (67).

## Sodium MRI in Neurological Disorders Other Than MS

First *in vivo* investigations using sodium MRI already stem from the 1980's: in an experimental model and in human investigations of stroke, Hilal and colleagues detected temporal changes of sodium levels over time. These studies already indicated the potential of sodium MRI as a biomarker for brain disorders (37, 46). However, technical restrictions limited brain sodium imaging to the investigation of widespread cerebral lesions or CSF (see above).

Yet, with the rapid development in scanner hardware and MRI software, several consecutive sodium MRI studies for stroke were conducted confirming highly elevated TSC in acute stroke due to estimated Na^+^/K^+^ ATPase breakdown, consecutive sodium accumulation, hypoxic cell death and perifocal edema (88). Furthermore, sodium MRI may represent a biomarker of viable, but hypoxic tissue-at-risk (“penumbra”) in stroke (88–90).

In primary brain tumors like low- and high-grade glioma, exaggerated proliferation rates lead to cellular membrane depolarization preceding cell division. Here, ^23^NaMRI may additionally be useful as a predictive biomarker for the discrimination of therapy responsive tissue (45, 77, 91–95).

Sodium MRI analysis of neurodegenerative diseases revealed whole-brain TSC increase in Huntington's disease independently of structural changes depicted by proton MRI. The caudate nucleus exhibited the highest TSC which correlated with gray matter atrophy and CAG repeat length (96). In addition, a small study in Alzheimer's disease (*n* = 5) reported a 7.5% brain TSC increase with an inverse correlation to hippocampal volume (97). Similarly, 9.4T sodium MRI of subjects with structural brain damage revealed loss of “cell volume fraction” (CVF) indicating a reduced CNS cell density. In contrast, individuals with a constant CVF may represent aging patients without disease. Hence, sodium MRI may evolve as a predictive biomarker for neurodegenerative diseases which are often hallmarked by early regional neuronal loss before the onset of clinical symptoms (98).

## Sodium MRI in Multiple Sclerosis (MS)

### Sodium MRI Alterations in Cerebral Lesions, NAWM, and NAGM in MS

**Figure 2:**
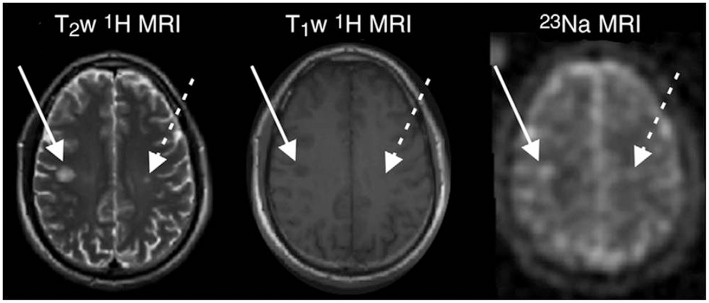
MR images in a 33-years-old man with early RRMS. Examples of substantial sodium accumulation in two macroscopic T2 lesions with two different signal intensity patterns at T1-weighted imaging: one lesion was hypointense (solid arrows) and one was isointense (dashed arrows) to normal-appearing white matter on T1-weighted image. Figure reproduced from (99) with permission of the Radiological Society of North America (RSNA).

In 2010, Inglese and colleagues published the first study applying ^23^NaMRI in 17 relapsing-remitting MS (RRMS) patients and 13 healthy controls, using a 3D radial gradient-echo UTE sequence at 3T (64). In MS, lesional (Figure 2) and gray-matter (GM) TSC was increased as compared to normal appearing white matter (NAWM). Further studies confirmed these findings (65, 99–101). As compared to healthy controls, the NAWM of MS patients exhibited an elevated TSC (mean 19.4 vs. 26.9 mM). This increase was particularly predominant in the cerebellum and splenium, yet without statistical significance. The normal-appearing gray matter (NAGM) displayed even higher sodium levels, but without any regional predominance (64). In this study, MS lesion analysis was restricted to plaques with a diameter >5 mm due to potential partial volume effects. The mean lesion sodium concentration was 35.3 mM, clearly higher than TSC of NAWM.

Gadolinium-enhancing acute MS lesions showed the highest TSC. This finding may be the direct consequence of inflammatory processes in the cell (e.g., mitochondrial failure, ATP deprivation, sodium accumulation) and the extracellular space (e.g., tissue damage, edema, enlarged extracellular space, infiltrating immune cells). However, analyses only comprised a low number of acute MS lesions and did not discriminate between intra- and extracellular sodium compartments. Therefore, it was not possible to determine the exact source of lesional sodium accumulation.

A study of Eisele et al. further analyzed neuroinflammatory aspects and studied the evolution of lesional sodium accumulation by ^23^Na MRI with a 3D radial sequence and SDWA at 3T. The authors analyzed acute and chronic lesions in 65 relapsing MS patients as compared to 10 controls (102). Mean TSC was quantified in all MS lesions with a diameter of >5 mm and in the NAWM as well as GM. First, TSC in NAWM and GM were higher in MS patients than in controls. Second, all types of MS lesions displayed a TSC increase. The most pronounced accumulation was seen in contrast-enhancing T1 lesions > T1 hypointense lesions > T1 isointense lesions. Interestingly, non-enhancing, hyperacute lesions with restricted diffusion on proton based diffusion-weighted MRI sequences showed a TSC comparable to the NAWM. Thus, TSC may not only serve as a biomarker for chronic tissue pathology and neurodegeneration, but also allow the detection and monitoring of inflammatory processes. Thus, this study further supported the use of TSC measured by sodium MRI as a potential biomarker for neuroinflammation. ^23^Na MRI may enable visualization of blood-brain barrier disruption without need for application of contrast-enhancing agents.

Another study used ^23^NaMRI in a case report on a large open-ring enhancing MS lesion. The authors reported intralesional sodium heterogeneity with declining TSC from the center of the active plaque (TSC: 50 mmol/l) across the enhanced periphery (33 mmol/l) toward the NAWM (26 mmol/l) (103). This gradual and centripetal TSC increase may result from the underlying degree of inflammation and mitochondrial dysfunction within acute MS lesions. Therefore, ^23^NaMRI may constitute a future biomarker for the extent of neuroinflammation. It may even point to inflammatory “tissue at risk” before persistent neuro-axonal damage occurs.

Our group additionally studied ^23^NaMRI in a case with an acute, enhancing tumefactive MS lesion over a follow-up of 5 weeks. Sodium accumulation outlasted contrast enhancement after steroid treatment as a potential sign of prolonged metabolic dysfunction and delayed recovery. At the same time, TSC in NAWM remained unaffected by steroid therapy (104).

However, comprehensive longitudinal studies reporting temporal evolution of sodium accumulation in acute MS lesions are still lacking. Upon repeated sodium MRI investigations in five healthy controls, WM and GM areas revealed a coefficient of variation for TSC <5% and an intra-class correlation coefficient of > 0.9. These data indicate sufficient reproducibility of ^23^NaMRI as a basis for future longitudinal studies (64).

Zaaroui and colleagues used a DA-3DPR sequence for sodium MRI at 3T to analyze 26 RRMS patients. They compared patients with a disease duration <5 vs. >5 years to healthy controls (99). The authors investigated TSC in three different compartments: GM, NAWM, and T2 lesions. In T2 lesions of all MS patients, TSC was higher than in WM of controls. In contrast, only the RRMS cohort with advanced disease duration exhibited a significantly increased TSC of GM and NAWM. Both MS groups displayed a similar TSC in T2 lesions and NAWM. Yet, GM TSC was higher in the advanced duration RRMS cohort. Nevertheless, this study was able to detect brain sodium accumulation even at the early stages of RRMS. When analyzing for anatomic distribution of TSC, the same study found widespread brain regions with elevated TSC in both MS cohorts. In advanced RRMS, TSC increase was scattered in the splenial, thalamic, cingular, parietal, frontal, and prefrontal cortices. A recent 7T study further complemented these findings of a widespread distribution of increased TSC in various MS GM and WM regions (101).

### Sodium MRI and MS Disease Course

Paling and colleagues conducted an investigation of 70 MS patients comprising three MS clinical subtypes (27 RRMS, 23 SPMS, 20 PPMS patients, and 27 controls). They applied a ramp sampled radial UTE sequence at 3 T with additional partial volume correction (65).

The authors analyzed TSC in cortical and deep GM, NAWM and in MS lesions, differentiated in T1 hypo- or isointense lesions. Independent of the disease course, MS patients exhibited an increased TSC of GM and NAWM as compared to controls. Additionally, deep GM and NAWM TSC were higher in the progressive MS subtypes. SPMS patients showed pronounced TSC in GM and NAWM as compared to RRMS. Further testing between MS subgroups was not significant. However, TSC of T1 hypointense lesions was higher in progressive MS subtypes than in RRMS. In conclusion, the study revealed increased sodium accumulation within MS lesions, NAWM and GM in all clinical MS courses. TSC accumulation was pronounced in SPMS and in patients with increased disability. This finding may serve as a hint for neuro-axonal damage, thus emphasizing the potential of ^23^NaMRI to provide a biomarker for neurodegeneration.

An investigation of Maarouf and colleagues included 20 progressive MS patients (11 PPMS, 9 SPMS) and 15 controls. The authors applied a DA-3DPR sequence at 3 T analyzing TSC of GM, NAWM and T2 lesions (100). They also found that TSC of T2 lesions and GM were significantly elevated in progressive forms as compared to controls. However, NAWM TSC was not significantly elevated in both progressive forms vs. controls. Independent of the analyzed brain tissue, no differences between PPMS and SPMS were detected. In this study, TSC accumulated to a higher degree in distinct brain areas of SPMS patients than in PPMS: Above all, it involved primary or supplementary locomotor areas consistent with the pronounced disability of patients with a median expanded disability status scale (EDSS) score of 5.5.

Interestingly, early sodium MRI studies and a study performed at 9.4 T did not find relevant age-dependent changes in TSC (64, 65, 98–100). However, a 7 T study described a positive correlation of age with WM and GM TSC as well as with GM intracellular sodium accumulation in healthy controls. In contrast, a negative association of age with intracellular sodium concentration (ISC) but not with extracellular sodium concentration (ESC) or TSC was detected in MS patients. The same study also showed correlations between disease duration, WM TSC and ISC for both, GM and WM as well as between extracellular sodium accumulation in GM and EDSS (101).

### Sodium MRI and MS Disability

In the first ever conducted MS sodium MRI study, Inglese and colleagues reported a low correlation of EDSS as a measure of disability with the mean TSC in T1-hypointense lesions (*r* = 0.22) as well as in NAWM and GM (*r* = 0.20). However, they did not find an association between disease duration, age or gender and TSC in lesions, GM or NAWM of RRMS patients (64).

Zaaroui et al. described no correlation of the TSC in T2 lesions or NAWM of RRMS patients with disability as measured by EDSS. However, GM TSC was significantly associated with EDSS as a potential biomarker for the degree of MS disability. In particular, the EDSS showed a positive correlation with the local TSC of the right primary motor area, middle frontal, and bilateral superior gyrus as well as the bilateral cerebellum (99).

In a study including progressive forms of MS, disability was correlated with TSC in deep GM and T1 isointense lesions. In addition, the authors showed independent associations of deep GM TSC with EDSS and features of the clinical assessment tool “Multiple Sclerosis Functional Composite” (MSFC). These data further support sodium MRI as a new method for monitoring disability and neurodegeneration (65).

In contrast, Maarouf et al. reported no significant correlation between TSC in T2 lesions or GM and EDSS or MSFC in their progressive MS study. Solely, the authors found an association between local TSC of the left premotor cortex and EDSS as well as of the left anterior prefrontal cortex and MSFC (100). Further brain regions of the limbic and the frontal areas displayed an increased TSC only in SPMS. Thus, the authors concluded that in PPMS, sodium accumulation was restricted to the motor system. In SPMS, it was more widespread involving regions related to higher cognitive functions.

### Sodium MRI and Correlation With Markers of Neurodegeneration

To date, brain atrophy is the “goldstandard” MRI marker. Yet, sodium MRI may provide additional information for imaging clinically relevant neurodegeneration (28, 105). Indeed, in the early 2010 study of Inglese and colleagues, RRMS patients already displayed a significantly lower normalized brain volume and GM volume. They also showed a trend toward lower WM volume as compared to controls.

TSC negatively associated with regional GM volume. However, there was no correlation of TSC with whole brain volume. TSC of NAWM did not correlate with any brain volume. In the respective control cohort, TSC in WM and GM showed an inverse correlation with normalized brain volume. Furthermore, TSC in RRMS associated with total T1 and T2 lesion volume (64).

An additional 3 T study showed an association of TSC in NAWM and GM with T2 lesion load. However, this study did not analyze brain atrophy (99). Application of 7 T ultra-highfield MRI showed no correlation of global and regional TSC, neither of intracellular or extracellular sodium concentrations to measures of brain volumes. In contrast, there was a trend for correlation of extracellular sodium accumulation and GM volume (101).

In 2017, Maarouf and colleagues investigated if brain TSC and GM atrophy were associated with cognitive dysfunction. They analyzed 58 RRMS patients in the early course and 31 controls using DA-3DPR at 3 T (106). The TSC increase in GM and NAWM was associated with cognitive dysfunction and predominantly located in neocortical regions. GM TSC even outmatched GM atrophy as a better predictor of cognitive dysfunction in MS patients. These data further emphasize the potential of sodium MRI for depiction of neurodegeneration, probably even at earlier stages than the “goldstandard” MRI brain atrophy. Hence, sodium MRI may show early neuronal dysfunction even before final neuronal damage occurs. Only the latter can be demonstrated by proton based MRI techniques (106).

To gain complementary information on microstructural pathologies, a recent analysis of 21 RRMS patients and 20 controls applied a DA-3DPR sodium MRI sequence at 3 T in combination with a proton based MR spectroscopy (proton echo planar spectroscopic imaging, 3D ^1^H-EPSI). Spectroscopy studies included N-acetyl aspartate (NAA; marker for mitochondrial activity), glutamate/glutamine (Glx; marker for neuro-astrocytic metabolism), total creatine (tCr; marker for cellularity), choline (Cho; marker for inflammatory demyelination) and myo-inositol (m-Ins; marker for glial activation) (107). TSC was elevated in all types of brain tissue in MS patients. MR spectroscopy revealed decreased Cho and Glx in GM, an increase of m-Ins but a decrease of NAA and Glx in NAWM and an increase in m-Ins but decrease in NAA in T2 lesions. In sum, TSC was negatively correlated with NAA as a marker for mitochondrial dysfunction and consecutive neuro-axonal damage. These data are consistent with findings from experimental studies pointing to mitochondrial damage as a consequence of toxic sodium accumulation (3, 108, 109). However, these studies did not correct for the influence of different sodium compartments.

### Sodium MRI and Differentiation of Intra- vs. Extracellular Sodium

In the early MS sodium MRI studies, the distinction of different sodium compartments was not possible, mainly due to limited MRI techniques. However, such a differentiation was regarded as highly relevant for a better understanding of MS pathogenesis. Yet, it remained unclear if elevated TSC was the result of rising extracellular fluid sodium due to edema, neuro-axonal damage or demyelination on the one hand, or the result of intracellular sodium accumulation due to inflammatory toxicity on the other (64, 65, 99).

The first study enabling differentiation of cellular compartments in 19 RRMS patients and 17 controls applied a combined single (SQ) and triple quantum filtered (TQF) 3D gradient echo ^23^NaMRI sequence at 7 T ultra-high field (101). The applied TQF technique used the different relaxation properties and signals of intracellular and extracellular distributed sodium ions. It thus enabled measurement of TSC, but also differentiation of the intracellular sodium concentration (ISC) and the intracellular sodium volume fraction (ISVF). ISVF is an indirect, inversely correlated measure of the extracellular sodium concentration (ESC): ISVF reduction is assumed to be a marker for a diminished intracellular volume and, accordingly, an increase of the extracellular space and ESC. As a limitation discussed by the authors, the applied model is based on the assumption, that the pathology itself does not change the ^23^Na relaxation times and that the TQF sequence enables a precise and unbiased sodium compartment differentiation. However, the quantitative accuracy of ISC measurements in MS patients is still undefined, since there is no non-invasive “goldstandard” that the ISC measurement can be compared to *in vivo*.

In accordance with previous 3 T studies, TSC of GM and MS lesions was higher than in WM and higher in MS than in healthy controls. ISC did not differ between the respective GM and WM but was higher in MS patients than in controls in GM and WM. In contrast, ISVF was lower in MS patients than in controls and higher in WM than in GM of both groups. In conclusion, TSC accumulation was in part depending on the growth of the extracellular compartment as potential consequence of axonal loss in MS. Nevertheless, it also depended on a distinct intracellular sodium increase. These results support findings of *ex vivo* and experimental studies suggesting a concomitant toxic metabolic dysfunction due to sodium imbalance (101).

Another study aimed at elucidating (1) differences in sodium levels between acute (= contrast enhancing) and chronic MS lesions and (2) differences between intracellular (ISC) and total sodium concentrations. Besides a regular DA-3DPR sequence, the authors also employed a fluid-attenuated sodium signal at 7 T in 29 MS patients (78). The applied fluid-attenuated sequence with a relaxation-weighted sodium signal preferentially depicts sodium ions with short relaxation times as found intracellularly. Thus, the setting enables a weighting toward the intracellular sodium compartment similar to previous approaches (4, 76, 77, 110). The study demonstrated that TSC and ISC were higher in acute as compared to chronic MS lesions. Hence, the fluid-attenuated sequence was useful to differentiate both types of lesions. TSC was positively correlated with T1 and T2 proton based lesion signals. In contrast, ISC only correlated with acute contrast enhancing T1 lesions. Interestingly, TSC and ISC levels were not associated. These data further support the additional biological significance of intracellular sodium accumulation measured by ^23^NaMRI. Thus, ISC increase may occur independently of extracellular sodium increase due to inflammatory edema or cell loss. This observation renders ISC a useful biomarker of metabolic neuroinflammatory processes.

In addition, this study contributed rare longitudinal sodium MRI data. Three patients were analyzed before and after steroid treatment indicating decrease of both sodium signals after treatment. Besides intracellular sodium accumulation, a distinct inflammatory hyper-cellularity may lead to elevated ISC in acute lesions. A combination of proton (lesion detection) and sodium (lesion differentiation) MRI may yield a neurodegenerative and neuroinflammatory biomarker and potentially an alternative to contrast agent application in the future (78).

Additionally, in the above mentioned case report of an acute enhancing MS lesion with open ring sign, also ISC was analyzed. ISC was reduced in the center of the acute lesion as compared to the periphery and NAWM. The low central ISC may be explained by enhanced cellular necrosis as compared to a more viable periphery. Thus, sodium MRI may constitute a useful biomarker for the degree of acute neuroinflammatory damage in MS (103).

### Sodium MRI and Further Fields of Applications in MS

Since low SNR and low spatial resolution is a major issue in sodium MRI, the incorporation of proton based anatomical MRI data in the reconstruction process enables improved image quality (111). A recent study described the application of an anatomically weighted second-order total variation (AnaWeTV) interative construction constraint in a MS patient including anatomical weighted MRI. AnaWeTV resulted in improved sodium MRI quality and less confounding partial volume effects, particularly in tissues or lesions that are visible in sodium and proton base MRI (60).

Another study was particularly engaged in the detection of potential errors of sodium MRI. Here, partial volume effects and spatially correlated noise artifacts impede quantification of sodium in small MS lesions (112). Besides a sodium-phantom analysis with given sodium concentration, sodium MRI signal variation in small lesions of five MS patients was compared to a computed predictive value using twisted projection imaging. Both, theoretical and *in vivo* sodium measurement pointed to a variation error of 20% in large, and even of 40–50% in small lesions as defined by the investigators. These data suggest underestimation of Na^+^ signals especially in small lesions and emphasize the limitations of sodium MRI despite improved imaging techniques.

Regular proton based MRI often requires gadolinium containing contrast agents. At the same time, several previous studies detected distinct MRI signal alterations of the dentate nucleus as a potential consequence of multiple gadolinium applications. Consequently, a recent study aimed at investigation of the dentate nucleus by sodium MRI at 3 T (113): in 12 MS patients and 6 controls, there was no difference in TSC between both groups despite a signal-altered dentate nucleus. These results suggest sustained tissue integrity of dentate nuclei with gadolinium deposition.

Finally, a recent study was able to exclude relevant influences of a preceding gadolinium application to the subsequent sodium MRI measurement. Despite a distinct quantitative influence of gadolinium on sodium relaxation times, this study further emphasized the compatibility and potential of combined proton and sodium MRI (114).

## Conclusions and Perspectives

Within the last decade, there was increasing evidence for the value of sodium accumulation measured by ^23^NaMRI as a biomarker for neurodegeneration and -inflammation in MS. Studies point to a widespread increase of TSC in MS as compared to healthy populations with a pronounced increase in the GM and in MS lesions as well as in progressive disease courses. Furthermore, sodium accumulation partly correlated with disability (as measured by EDSS) and brain atrophy as the proton based MRI “goldstandard” for monitoring neurodegeneration in MS. Moreover, the TSC increase occurs even in “unaffected” NAWM as defined by standard proton based MRI. Thus, TSC as measured by sodium MRI is discussed as an early biomarker of neurodegenerative changes in MS brains.

The value of ^23^NaMRI as a potential tool for monitoring of neuroinflammation has mainly been restricted to lesional TSC measurements. These studies consistently showed highest TSC in acute contrast-enhancing lesions as compared to NAWM. To date, the investigation of intra- vs. extracellular sodium accumulation in inflammatory lesions is limited to case reports or studies including very few MS patients.

Moreover, the analysis of acute MS lesions still necessitates a large lesion size of roughly >5 mm to minimize partial volume effects. Furthermore, large longitudinal studies to examine the temporal evolution of the sodium content in MS lesions and the correlation to conventional markers of inflammation are still lacking. Meanwhile, novel imaging techniques allowing for discrimination of tissue compartments are in part capable to delineate increased extracellular sodium. These studies analyze the expanded extracellular space as consequence of neuro-axonal damage or inflammatory edema caused by increased intracellular sodium due to intraneuronal/-axonal sodium accumulation with its consecutive toxic intracellular signal cascades (4, 67, 78, 101). Preceding findings indicate that ISC in MS are elevated in different brain regions as compared to healthy controls. However, TSC accumulation in MS was shown to depend on both, ESC and ISC increase. These findings suggest an expanded extracellular compartment i.e., due to axonal loss in MS on the one hand, but also on a distinct intracellular accumulation on the other. However, the precise differentiation of intra- vs. extracellular sodium via ^23^NaMRI is still limited. In particular, a mutual influence of intra- and extracellular spaces on each other cannot be definitely excluded. Progress in the development of respective imaging techniques will enable a more detailed insight in the diverse origin and effects of intra- vs. extracellular sodium accumulation.

Despite all progress, sodium MRI still has to overcome several limitations: initial studies displayed huge range of TSC quantification of more than 40 mM, mainly due to different scanner hardware, acquisition protocols or quantification models. Novel sodium imaging techniques improved the quantification range to roughly 10 mM but still may vary significantly (39). Thus, comparability of results from different study groups is complicated. Using the intraventricular CSF sodium signal as an intra-individual reference signal for quantification of sodium concentrations was recently discussed to specify sodium measurement. This approach is based in the observation that sodium levels in the CSF may be stable at the levels of extracellular fluid i.e., 140 mM (66, 115).

Yet, as a consequence of the physical properties of Na^+^, application of ultra-high field ≥7 T scanners and/or long acquisition times is warranted for “state-of-the-art” sodium MRI. This is of particular relevance when aiming at the precise differentiation of sodium compartments.

In consequence, further development of sodium MRI techniques and hardware is crucial (1) to improve SNR and resolution, (2) to diminish partial volume effects and scanning times, and (3) to enable precise differentiation of sodium compartments.

The future technical improvement together with the demonstrated high potential of brain sodium as a biomarker in neurological disorders may pave the way for the implementation of sodium MRI in clinical routine. The implementation of this ambitious goal may be further supported by affordable sodium MRI head coils and software packages enabling widespread sodium measurement at commercial MRI systems (39).

Since ^23^NaMRI requires no contrast agents, similar contraindications as for conventional proton MRI apply. Even at ultra-high field strengths, MRI is well-tolerated, thus further supporting an extended application of ^23^NaMR investigations (29, 68, 116). However, none of the published studies so far comprised an MS collective with *n* > 100 thus in part limiting their significance. In consequence, multi-center studies with strictly defined MS patient cohorts and sodium MRI methods are warranted to improve the validity of future studies.

Furthermore, improved sodium MRI techniques may enable the future investigation of smaller regions of interest i.e., inflammatory brain lesions with a diameter <5 mm or spinal cord sodium concentrations. Analyzing the spinal cord would add useful information about disability-relevant MS pathology beyond previous *ex vivo* or proton based MRI analyses (117).

As another attractive location for sodium MRI, studies of hypertension, renal and rheumatological diseases displayed elevated sodium deposition in the skin and muscle (118–120). Together with experimental findings of proinflammatory properties of elevated sodium levels in the skin, analysis of dermal and muscular soft tissue sodium could also be interesting in the field of MS. Here, the inflammatory pathogenesis may likely be initiated in the periphery before immune cells enter the CNS (23). Hence, sodium MRI may help to elucidate sodium dependent effects of the yet scarcely characterized origin of inflammatory processes in the periphery of MS patients.

Finally, sodium MRI has not been applied in other acute or chronic inflammatory diseases of the CNS, such as acute disseminated encephalomyelitis (ADEM), vasculitis, granulomatous diseases, or aquaporine-4-antibody associated neuromyelitis optica spectrum diseases (NMO-SD). Here, the technique may add further valuable insights beyond conventional proton MRI which often cannot sufficiently differentiate between these entities (28). In addition, the effects of underlying disease-modifying therapies on MRI brain sodium levels have not been analyzed yet and remain to be demonstrated. Such studies warrant longitudinal investigations of MS patients under immunomodulatory treatment.

Combination of sodium MRI with additional imaging tools beyond standard proton MRI may gain novel information about pathological metabolic processes associated with sodium accumulation. Here, additional MRI techniques [i.e., myelin water imaging (MWI), magnetization transfer ratio (MTR), diffusion tensor imaging (DTI), magnetic resonance spectroscopy (MRS), optical coherence tomography (OCT) or metabolic imaging techniques, such as positron emission tomography (PET)] would be promising candidates (48).

In conclusion, modern sodium MRI has in part overcome its inherent physical limitations, but still is in need for further development. With its capability to give yet unknown insights in the pathology of MS, this imaging technique deserves further investigation aiming at implementation of sodium accumulation as a biomarker for neurodegeneration and -inflammation in the future.

## Data Availability Statement

No primary datasets were generated for this study. KH and AN have access to referenced articles and are responsible for all data presented in the manuscript.

## Author Contributions

KH drafted the work, contributed to the conception and interpretation of the work, and acquired the included data and references. TE contributed to the conception and interpretation of the work and revised it critically for important intellectual content. RL contributed to drafting the work, contributed to the conception and interpretation of the work, and revised it critically for important intellectual content. AN contributed to drafting the work, acquired the included data and references, contributed to the conception and interpretation of the work, and revised it critically for important intellectual content. All authors provide approval for publication of the content.

### Conflict of Interest Statement

The authors declare that the research was conducted in the absence of any commercial or financial relationships that could be construed as a potential conflict of interest. The reviewer AH declared a past co-authorship with one of the authors RL to the handling Editor.

## Abbreviations

ADEM: acute disseminated encephalomyelitis
AnaWeTV: anatomically weighted second-order total variation
ATP: adenosine triphosphate
ATPase: adenosine triphosphatase
Ca^2+^: calcium
CAG, trinucleotide of cytosine, adenine: guanine
cho: choline
^35^Cl: chlorine
CNS: central nervous system
CSF: cerebrospinal fluid
CVF: cell volume fraction
DA-3DPR: density-adapted 3D projection reconstruction
DTI: diffusion tensor imaging
EAE: experimental autoimmune encephalomyelitis
EDSS: expanded disability status scale
ESC: extracellular sodium concentration
^19^F: fluorine
FLAIR: fluid-attenuated inversion recovery
Glx: glutamate/glutamine
GM: gray matter
H^+^: Proton hydrogen
H-EPSI: proton echo planar spectroscopic imaging
IR: inversion recovery
ISC: intracellular sodium concentration
ISVF: intracellular sodium volume fraction
^39^K: potassium
K^+^: potassium
l: liter
m-Ins: myo-inositol
mM: millimolar
mm: millimeter
MQF: multiple quantum filtering
MRI: magnetic resonance imaging
MRS: magnetic resonance spectroscopy
MS: multiple sclerosis
ms: millisecond
MSFC: multiple sclerosis functional composite
MTR: magnetization transfer ratio
MWI: myelin water imaging
n: number
^23^Na: sodium
Na^+^: sodium
NAA: N-acetyl aspartate
NaCl, sodium chloride: salt
NAGM: normal-appearing gray matter
Nav: voltage gated sodium channel
NAWM: normal-appearing white matter
NMO-SD: neuromyelitis optica spectrum diseases
^17^O: oxygen
OCT: optical coherence tomography
^31^P: phosphorous
PET: positron emission tomography
PPMS: primary progressive multiple sclerosis
*r*: correlation coefficient
RF: radiofrequency
ROI: region of interest
RRMS: relapsing remitting multiple sclerosis
SDWA: sampling density weighted apodization
SNR: signal-to-noise ratio
SPMS: secondary progressive multiple sclerosis
SQF: single quantum filtering
T: Tesla
T1: T1-weighted MRI sequence
T2: T2-weighted MRI sequence
tCr: total creatine
Th: T-helper cell
TSC: tissue sodium concentration
TPI: twisted projection imaging
TQF: triple quantum filtering
UTE: ultra-short echo time
WM: white matter.
